# TbKINX1B: a novel BILBO1 partner and an essential protein in bloodstream form *Trypanosoma brucei*

**DOI:** 10.1051/parasite/2022015

**Published:** 2022-03-09

**Authors:** Doranda Perdomo, Elodie Berdance, Gertrud Lallinger-Kube, Annelise Sahin, Denis Dacheux, Nicolas Landrein, Anne Cayrel, Klaus Ersfeld, Mélanie Bonhivers, Linda Kohl, Derrick R. Robinson

**Affiliations:** 1 University of Bordeaux, CNRS, Microbiologie Fondamentale et Pathogénicité, UMR 5234 33000 Bordeaux France; 2 Department of Genetics, Bldg. NW1, University of Bayreuth, Universitätsstraße 30 95440 Bayreuth Germany; 3 Institut Polytechnique de Bordeaux, Microbiologie Fondamentale et Pathogénicité, UMR 5234 33000 Bordeaux France; 4 UMR 7245 Molécules de Communication et Adaptation des Micro-organismes, Muséum National d’Histoire Naturelle, CNRS, CP52 61 rue Buffon 75231 Paris Cedex 05 France

**Keywords:** Basal bodies, Cytoskeleton, Trypanosome, Kinesin, BILBO1, Flagellar pocket collar

## Abstract

The flagellar pocket (FP) of the pathogen *Trypanosoma brucei* is an important single copy structure that is formed by the invagination of the pellicular membrane. It is the unique site of endo- and exocytosis and is required for parasite pathogenicity. The FP consists of distinct structural sub-domains with the least explored being the flagellar pocket collar (FPC). TbBILBO1 is the first-described FPC protein of *Trypanosoma brucei.* It is essential for parasite survival, FP and FPC biogenesis. In this work, we characterize TbKINX1B, a novel TbBILBO1 partner. We demonstrate that TbKINX1B is located on the basal bodies, the microtubule quartet (a set of four microtubules) and the FPC in *T. brucei*. Down-regulation of TbKINX1B by RNA interference in bloodstream forms is lethal, inducing an overall disturbance in the endomembrane network. In procyclic forms, the RNAi knockdown of TbKINX1B leads to a minor phenotype with a small number of cells displaying epimastigote-like morphologies, with a misplaced kinetoplast. Our results characterize TbKINX1B as the first putative kinesin to be localized both at the basal bodies and the FPC with a potential role in transporting cargo along with the microtubule quartet.

## Abstract (fr)

La poche flagellaire (PF) de l’agent pathogène *Trypanosoma brucei* est une structure importante à copie unique formée par l’invagination de la membrane pelliculaire. Elle est le site unique de l’endo- et de l’exocytose et est nécessaire à la pathogénicité du parasite. La PF est constituée de sous-domaines structurels distincts, le moins exploré étant le collier de poche flagellaire (CPF). TbBILBO1 est la première protéine du CPF décrite. Elle est essentielle pour la survie du parasite et la biogenèse de la PF et du CPF. Dans ce travail, nous caractérisons TbKINX1B, un nouveau partenaire de TbBILBO1. Nous démontrons que TbKINX1B est localisée au niveau des corps basaux, du quartet de microtubules (un ensemble de quatre microtubules) et du CPF chez *T. brucei*. La diminution de l’expression de TbKINX1B par ARN interférence dans les formes sanguines est létale, induisant une perturbation globale du réseau endomembranaire. Dans les formes procycliques, l’ARN interférence conduit à un phénotype mineur avec un petit nombre de cellules présentant des morphologies de type épimastigote, avec un kinétoplaste mal placé. Nos résultats caractérisent TbKINX1B comme la première kinésine putative à être localisée à la fois au niveau des corps basaux et du CPF avec un rôle potentiel dans le transport de cargaison le long du quartet de microtubules.

## Introduction

*Trypanosoma brucei* is a zoonotic pathogen and the etiological agent of sleeping sickness with 60 million people living in areas with a high risk of infection, even though fewer than 2000 cases are detected per year. The procyclic form (PCF) and bloodstream form (BSF) *T. brucei* cell has a polarized organization and importantly, within its posterior end, it houses a unique structure called the flagellar pocket (FP) from which the flagellum emerges. This organelle-like structure is the exclusive site for endo- and exocytotic activity [32].

Within eukaryotic cells, transport of material often involves the activity of microtubule-dependent processes mediated by molecular motors such as kinesins (KIN) or dyneins. While most kinesins transport cargo through their interaction with microtubules (MT) by generating force through ATP hydrolysis [24, 25], some of these motor proteins depolymerize MTs and participate in regulating MT dynamics [33]. A comprehensive phylogenetic analysis performed in a wide range of eukaryotes has allowed a revised classification of kinesins. Currently, there are 17 kinesin families (kinesin-1 to -17), as well as 14 additional paralog groups that cannot be assigned to any of the kinesin families (kinesinX1-14). Little information is available for the paralog groups, as they contain a few members [45]. Trypanosomes possess a large number of kinesin encoding genes (37 genes in *T. brucei* plus 9 highly divergent genes versus 8 plus 1 highly divergent in *Plasmodium falciparum* [44] and 30 in *Homo sapiens*), indicating that some of these proteins could play essential and specific roles in these parasites or be redundant [45]. Functional characterization of kinesins in *T. brucei* has shown multiple, distinct and essential roles in flagellar construction, cell morphology, organelle segregation and mitosis [20, 27, 12, 13, 45].

We are interested in the organisation of the flagellar pocket and associated structures which ensure tight cellular and molecular links between the endomembrane components, in particular the flagellar pocket collar (FPC) and the hook complex [34, 36]. We previously identified and characterized the first FPC member, BILBO1 [9, 21, 41, 42] and demonstrated that down-regulation of *BILBO1* by RNA interference (RNAi) prevents the formation of a new FP and FPC, and is lethal in both PCF and BSF cells [9]. To identify other FPC components, we used the structural information for BILBO1 to design a yeast-two hybrid (Y2H) genomic screen, using BILBO1 as bait. Using this protocol, we identified several partner proteins and among these is the kinetoplastid-specific putative kinesin Tb927.7.3000 (initially named FPC5 [21]). Tb927.7.3000 protein belongs to the same kinetoplastid-specific KINX1 clade [45] as TbKINX1, a flagella connector protein (also named FCP2) [40]. Tb927.7.3000 contains a typical kinesin sequence motif and is now referred to as TbKINX1B, according to the kinesin nomenclature [45]. The Y2H screen identified amino acids (aa) 517–715 as the BILBO1-binding domain (B1BD). We further showed that this interaction with BILBO1 was dependent on the two BILBO1 EF-hand calcium-binding motifs [21].

TbKINX1B is part of the cytoskeleton of the trypanosome and is localized at the basal bodies as well as the FPC area, similar to BILBO1. RNAi down-regulation of TbKINX1B expression shows little effect in PCF but is lethal in BSF, where abnormal cells with an enlarged flagellar pocket are seen. Intriguingly, the absence of TbKINX1B does not affect the localization of TbBILBO1 in both forms.

We propose that TbKINX1B could be involved in the transport of cargos from the basal bodies to the FPC along the MTQ.

## Material and methods

### Ethics

The use of animals for the generation of monoclonal antibodies was in accordance with the rules of the ethical committee of the University of Bayreuth and licensed by the Government of Lower Franconia (licence RUF-55.2.2-2532-2-12-46-13).

### Cell culture

The PCF *T. brucei* 427 29.13 and BSF *T. brucei* 427 90.13 cell lines (named wild-type WT used as controls) both co-expressing the T7 RNA polymerase and tetracycline repressor were cultivated with the appropriate antibiotics and transfected as specified in [21]. The *TbBILBO1*^*RNAi*^ cell line was previously described in [9]. All tetracycline inductions were carried at 10 μg/mL.

### Purification of TbKINX1B motor domain proteins

Wild type motor domain (MD) of TbKINX1B (TbKINX1B^MD^) and mutant TbKINX1B–ΔP-loop^MD^, were cloned into pET32c vector for expression in frame with the coding sequence of N-terminal thioredoxin-6 histidine in *E. coli* BL21(*DE3*) using primers: 5′–tgtgtcgacaaatgacgtctcaaacgtcg–3′/5′–tgtgcggccgctcagcgctggtcttcgttgac–3′. Deletion of the P-loop domain (TbKINX1B–ΔP-loop^MD^) was achieved by direct mutagenesis (QuickChange kit, Agilent), following the manufacturer’s recommendations and with the use of the following primers: 5′–tcatgtttgtttgcgtactacagcatgattgggccc–3′/5′–gggcccaatcatgctgtagtacgcaaacaaacatga–3′. Protein expression was induced for 1 h at 37 °C with 1 mM isopropyl-b-thiogalactopyranoside (IPTG). Cells were harvested by centrifugation at 4000 ×*g* for 20 min and the pellet resuspended in binding buffer (20 mM Tris-HCl pH 7.4, 150 mM NaCl, 5% Glycerol, 5 mM imidazole supplemented with 1 mM PMSF (phenylmethylsulfonyl fluoride) and Protease inhibitor cocktail set III-EDTA-free (Calbiochem), and 200U Benzonase) and lysozyme 0.1 mg/mL). The mix was left 30 min on ice. Cells were lysed by sonication and the lysate was centrifuged at 10,000 ×*g* for 20 min at 4 °C. Soluble recombinant proteins were loaded onto a His FF HiTrap column (GE Healthcare) and washed in binding buffer supplemented with 20 mM imidazole. Finally, the proteins were eluted with a 35–300 mM imidazole gradient in binding buffer. The same protocol was applied for the purification of the N-terminal thioredoxin-6 histidine tag (Trx-6His), which was used as a control in the Kinesin ATPase activity assay. Three independent purifications of each recombinant protein were performed and each was used for the Kinesin ATPase activity.

### Production of anti-TbKINX1B mouse monoclonal

The C′-terminus of TbKINX1B (aa 821–1342) was expressed in *E. coli* XL1Blue bacteria, using the pTrcHis vector (Invitrogen). This truncation contained an N-terminal His-tag. The protein was purified under native conditions on NI-NTA columns (Qiagen) and used to inject three BALB/c mice. Mice were inoculated with the purified protein as follows: first injection: 50 μg in PBS mixed with complete Freund’s adjuvant, intraperitoneally (IP). Second and third injections: 25 μg mixed with incomplete Freund’s Adjuvant (IP). Fourth injection, 25 μg in PBS, (IP). Injections were done at intervals of three weeks and the mouse with best serum response by ELISA assays was used for myeloma fusion. Fusion of spleen and myeloma cells (P3X63-Ag8.653), was PEG-induced. Screening of hybridoma culture supernatants was done initially by ELISA assay, and positive clones were then screened by Western blot and immunofluorescence. The resulting antibody is an IgM (kappa light chain). Supernatants were collected and precipitated with 50% ammonium sulphate.

### Kinesin ATPase activity

The ATPase activity of purified proteins (TbKINX1B^MD^, TbKINX1BΔP-loop^MD^ and TbKINX1BTrx-6His) was evaluated using the commercially available Enzyme Linked Inorganic Phosphate Assay (ELIPA, Cytoskeleton). The assay was done with the GST recombinant human kinesin heavy chain motor domain (KHC^MD^) as a positive control in presence of MTs (+MTs), and as a negative control in absence of MT (−MTs) (Cat. #KR01, Cytoskeleton Inc.), and in the presence of taxol-stabilized microtubules (MT002, Cytoskeleton Inc.). Each condition (microtubules (MT) alone, kinesin alone and microtubules + kinesin) was performed in triplicate. Kinetic read-out was initiated upon the addition of ATP and followed using an OPTIMA plate reader at a fixed wavelength of 360 nm of absorbance. Proteins (including positive control) were used at 0.4 nM. The rate of ATPase, as nM per minute per mg of protein was calculated using a standard Pi curve, according to the manufacturer’s recommendations. Independent experiments from independent protein batches purifications (*n* = 3) were used and the results were plotted using Prism Software, Version 5.

### Procyclic TbKINX1B RNA interference (*TbKINX1B*^*RNAi*^) cell line

A fragment of *TbKINX1B* coding sequence (bp 2533–2941) was amplified using oligonucleotides: 5′–ctcgagggaaaagcagcaacgacttc–3′ and 5′–aagcttactcacgctccatctcgact–3′. The PCR product was cloned into the double T7 promoter pZJM vector [2]. The construct was linearized with *Not*I, and PCF cells were transfected by electroporation. Clonal transfectants were selected with phleomycin 5 μg mL^−1^ followed by serial dilution for clonal selection.

### Bloodstream form TbKINX1B RNA interference (*TbKINX1B*^*RNAi*^) cell line

Bloodstream form cells *Tb*427.90.13 were transfected by electroporation with 10 μg of *Not*I linearized plasmid and selected with 2.5 μg mL^−1^ phleomycin 24 h post-transfection, followed by serial dilution for clonal selection.

### TbKINX1B plasmid constructs in procyclic cells

Full-length (TbKINX1B), motor domain (TbKINX1B^MD^) and the BILBO1 binding domain (TbKINX1B^B1BD^) were amplified by PCR using primers pairs: 5′–tgtaccggtatgacgtctcaaacgtcg–3′/5′–tgttctagatccctccccgtcgataca–3′, 5′–tgtaccggtatgacgtctcaaacgtcg–3′/5′–tgttctagagcgctggtcttcgttgac–3′, 5′–tctaccggtatgcaggtagaggaggggagg–3′/5′–tgttctagactctacccgacgctctaa–3′, respectively. The PCR products were cloned into the vector pCR^®^-Blunt II-TOPO (Life Technologies), according to the manufacturer’s instructions. Constructs were digested by *Age*I and *Xba*I, and sub-cloned into digested pLew100-X-3myc vector [15] for C-terminal myc-tagged fusion protein. Constructs were verified by sequencing. PCF cells were transfected by electroporation and selected with 5 μg mL^−1^ phleomycin 24 h post-transfection. A clonal cell line was obtained by limiting dilution.

### Immunofluorescence

PCF detergent-extracted cells and permeabilized whole cells were prepared as described in [1] and fixed in 3% paraformaldehyde (PFA). The following primary antibodies were used diluted in PBS, supplemented with 0.1% Tween 20 (PBS-Tween 0.1%): (anti-BILBO1 rabbit polyclonal 1:6000), anti-TbKINX1B (home-made mouse IgM mAb specific to aa 820–1324, 1:4000), anti-FTZc (1:1000, [10]), anti-PFR2 (L8C4 1:10, [20]), and YL1/2 (Chemicon, MAB1864). Commercial secondary antibodies were secondary anti-mouse-FITC (Sigma F2012, 1:100), anti-rabbit Alexa 594 (Molecular Probes A11012, 1:100), anti-rat Alexa 488 or Alexa 594-conjugated for YL1/2 (Molecular Probes A21470 or 21471, dilution, 1:100). DNA was stained using DAPI. Images were acquired with Metamorph software on a Zeiss Imager Z1 microscope and processed by ImageJ. In the case of cells that were used for quantification of the phenotypes, BSF or PCF were fixed in methanol for 30 min and further used for immunofluorescence using PFR2 marker L8C4. Acquisition of images using 63× objective and random slide auto-focus application was employed using Metamorph software. A minimum of 200 cells in each case was counted, in three independent experiments (*n* = 3) and results were plotted using Prism Software, Version 5.

### Electron microscopy

Log-phase BSF control and TbKINX1B^RNAi^ 48 h induced cells were fixed by adding fixatives directly to medium to a final concentration of 2.5% glutaraldehyde for 10 min, cells were collected and processed as in [37].

### Immuno-electron microscopy

Log-phase *T. brucei* PCF cells were harvested by centrifugation 800 ×*g* for 10 min (min) and resuspended in 500 μL PBS on a clean sheet of Nescofilm. The cells were then adsorbed onto glow-discharged Formvar and carbon-coated grids for 15 min room temperature (RT). To prepare extracted flagella, cells on grids were first detergent-extracted in PEME buffer (100 mM PIPES, 1 mM MgSO4, 0.1 mM EDTA, 2 mM EGTA, pH6.9) plus 1% NP-40 (15 min, RT), rinsed with PEME, then further extracted in PEME buffer, 1% NP-40, 1 M KCl (20 min, 4 °C). Grids were then rinsed four times (5 min, RT) in PEME buffer and fixed in 3% PFA in PEME buffer (5 min, RT) then rinsed four times (5 min, RT) in 100 mM glycine in PBS. After extraction and rinsing with glycine buffer as described above, grids were moved through 2 × 10 min drops of blocking buffer (PBS with 1% fish skin gelatin and 0.1% Tween 20). Grids were then incubated with 25 μL of mAb (TbKINX1B diluted 1:50 and, if indicated in the figure, combined with rabbit polyclonal TbBILBO1 1:2000 [1]), in blocking buffer for 2 h at RT. After primary incubation, grids were blocked 4 times, 5 min each, as described above. Then, grids were incubated with goat anti-mouse IgM 15 nm gold conjugated secondary antibody diluted 1:50 (British Biotech, GAMM15) and if TbBILBO1 was used with protein A/G mix 6 nm gold conjugated (Electron Microscopy Sciences). Grids were then transferred on a drop of blocking solution 4 times 5 min in PBS, then fixed in 2.5% glutaraldehyde and negatively stained in 10 μL of 5% NanoVan (Methylamine Vanadate-Nanoprobes). Samples were viewed on a MET FEI TECNAI 12 TEM electron microscope.

### Immunoprecipitation

*Trypanosoma brucei* 427 29.13 PCF cells were grown up to log phase. The input material for IP (8 × 10^8^ cells) was washed in ice-cold phosphate buffer saline (PBS) and resuspended in 2 mL of lysis buffer (150 mM NaCl, 1 mM DTT, 1% NP-40 (Igepal CA-630), 25 mM Tris-HCl, pH 7.6) complemented with complete protease inhibitors (Roche) and 1 mM PMSF. The lysate was left on ice for 15 min and then sonicated (Bioruptor Plus) for 5 cycles of 30 s, level 5 amplitude. The lysate was cleared by centrifugation (30 min at 5000 ×*g*) and soluble material was used for the IP. A total of 50 μL of Dynabeads-Protein G (Life Technologies) were cross-linked to TbKINX1B rabbit polyclonal antibody (Eurogentec) or an unspecific antibody rabbit α-myc polyclonal antibody, using dimethyl 3,3′-dithiobispropionimidate (DTBP, Thermo Scientific), according to the manufacturer’s instructions. Soluble material was incubated with the beads overnight at 4 °C, in batch. Unbound material was kept for protein analysis (Flow-through, FT fraction), as well as the washes performed with the lysis buffer (W fraction). Beads were resuspended in 2× Laemmli buffer for 20 min at room temperature and then boiled for 10 min. Analysis of each of the fractions was carried by SDS-PAGE and immunoblot using mouse anti-TbKINX1B and rabbit anti-BILBO1 antibodies.

### Western blotting

Whole cells or detergent-extracted cytoskeleton (CSK) samples were prepared as previously described [9] with a concentration of 1 × 10^7^ or 2 × 10^7^ (BSF or PCF) cells/well, as mentioned according to the experiments. Whole cell (T), CSK-enriched pellets (P), and soluble proteins (S) were prepared as described before [21], with the addition of 1% NP-40 and 150 mM NaCl. Proteins were separated on SDS-PAGE and transferred onto 0.2 μm PVDF membrane. The following primary antibodies were used in the indicated cases: α-BILBO1 (pAb 1:200), α-TbKINX1B (rabbit polyclonal 1:2000 or mouse monoclonal 1:1000), α-Tubulin (TAT1, 1:1000, [34]), anti-enolase (1:10,000), and α-His (Sigma H-1029, 1:3000). Incubations with secondary antibodies goat α-rabbit HRP (Jackson 115-055-068) and goat α-mouse HRP (Jackson 115-035-044) were also performed as cited above. Labelling was revealed with Clarity ECL Substrate (Bio-Rad) and revealed using ImageQuant LAS400. Quantifications were performed using ImageJ. Errors bars in graphs represent the standard error (*n* = 3).

## Results

### TbKINX1B is a putative N-kinesin with ATPase activity

The gene Tb927.7.3000 [6] was previously identified in a genomic yeast two-hybrid library screen with TbBILBO1 as bait (Hybrigenics) [21] and codes for a putative-kinesin (named here TbKINX1B) that belongs to the paralog group, Kinesin-X1 of kinesins, as classified by Wickstead et al. [45].

**Figure 1:**
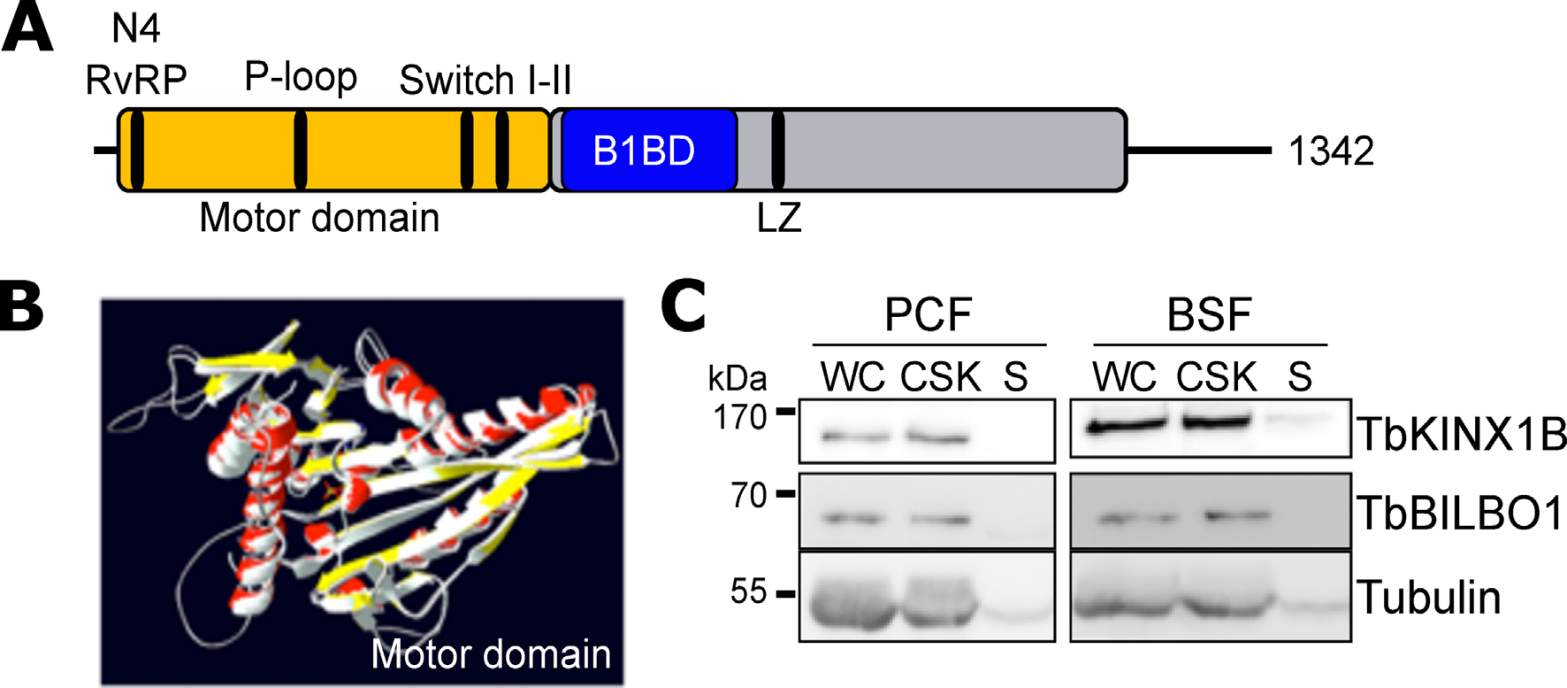
*TbKINX1B is an N-kinesin with a classical motor domain*. A. Predicted secondary structure representation of TbKINX1B protein. The kinesin motor domain (TbKINX1B^MD^ aa. 4-496 with P-loop aa. GQTGSGKT, Switch I aa. NEHSSRSH and Switch II aa. DLAGSE), TbKINX1B leucine zipper (LZ, aa. 756–784), the coiled-coil (aa. 1082–1297), and the TbBILBO1-binding domain (B1BD aa. 517–715) are shown. B. Alignment-based homology model of TbKINX1B motor domain to *Mus musculus* KIF1A motor domain (PDB 1I5S) by Phyre2 software. The predicted tertiary structure is represented as a superposition of both kinesin motor domains, TbKINX1B (colored: yellow for β-sheet and red for α-helix) and KIF1A (white), corresponding to 100% match. C. Western blot analysis of TbKINX1B in PCF and BSF cells. TbKINX1B, TbBILBO1 and Tubulin were probed on whole cells (WC), detergent-extracted cytoskeleton (CSK) and soluble fraction (S) of lysed cells.

TbKINX1B is a 1342 amino acids protein with a predicted molecular mass of 151.3 kDa. *In silico* characterization of the primary and secondary structure of TbKINX1B (Fig. 1A) identified an N-terminal motor domain (aa 4–496) with the predicted four motifs involved in nucleotide-binding: the P-loop (GxxxxGKT/S) for phosphate-binding loop, the N2 or Switch-I (NxxSSRS), the N3 or Switch-II (DLAGxE), and N4 (RvRP) motif [39] (Fig. 1A). The central region of TbKINX1B contains a coiled-coil domain (aa 501–862) including a leucine zipper motif (aa 756–784) and the aa 517–715 domain, identified in the Y2H screen as the TbBILBO1-binding domain (B1BD) [21]. Finally, a coiled-coil region (aa 952–1304) is present in the C-terminal part of the protein. Tertiary structural organization of TbKINX1B was obtained by submitting the full-length protein sequence to Phyre2 [29] for an alignment-based homology model. Results showed that full-length TbKINX1B shares 48% structural identity with *Mus musculus* KIF1A (PDB 1I5S, N-terminal kinesin) with 100% confidence in the proposed motor domain model (Fig. 1B).

In order to test TbKINX1B ATP hydrolysis activity *in vitro* when binding to MTs, we affinity purified the recombinant TbKINX1B motor domain (aa 4–496, _Trx-6His_TbKINX1B^MD^) and the motor domain deleted of its P-loop motif (_Trx-6His_TbKINX1B-ΔP-loop^MD)^. The proteins were expressed with a thioredoxin N-terminal tag and a 6-histidine tag (_Trx-6His_TbKINX1B^MD^, _Trx-6His_TbKINX1B-ΔP-loop^MD^) in *Escherichia coli* and affinity purified (Fig. S1A). The TbKINX1B^MD^ protein hydrolyses ATP with a Vmax of 288 nMol of ATP hydrolyzed per minute per mg of kinesin (nM × min^−1^ × mg^−1^). Unfortunately, the purified motor domain was prone to degradation (Fig. S1B), which could explain the reduced activity measured. As expected, no ATPase activity was detected for the motor domain deleted of the P-loop motif (_Trx-6His_TbKINX1B-ΔP-loop^MD^). Taken together, these results indicate that TbKINX1B displays the hallmarks of a typical N-kinesin.

### TbKINX1B localises to the basal bodies, the MTQ and the FPC

**Figure 2:**
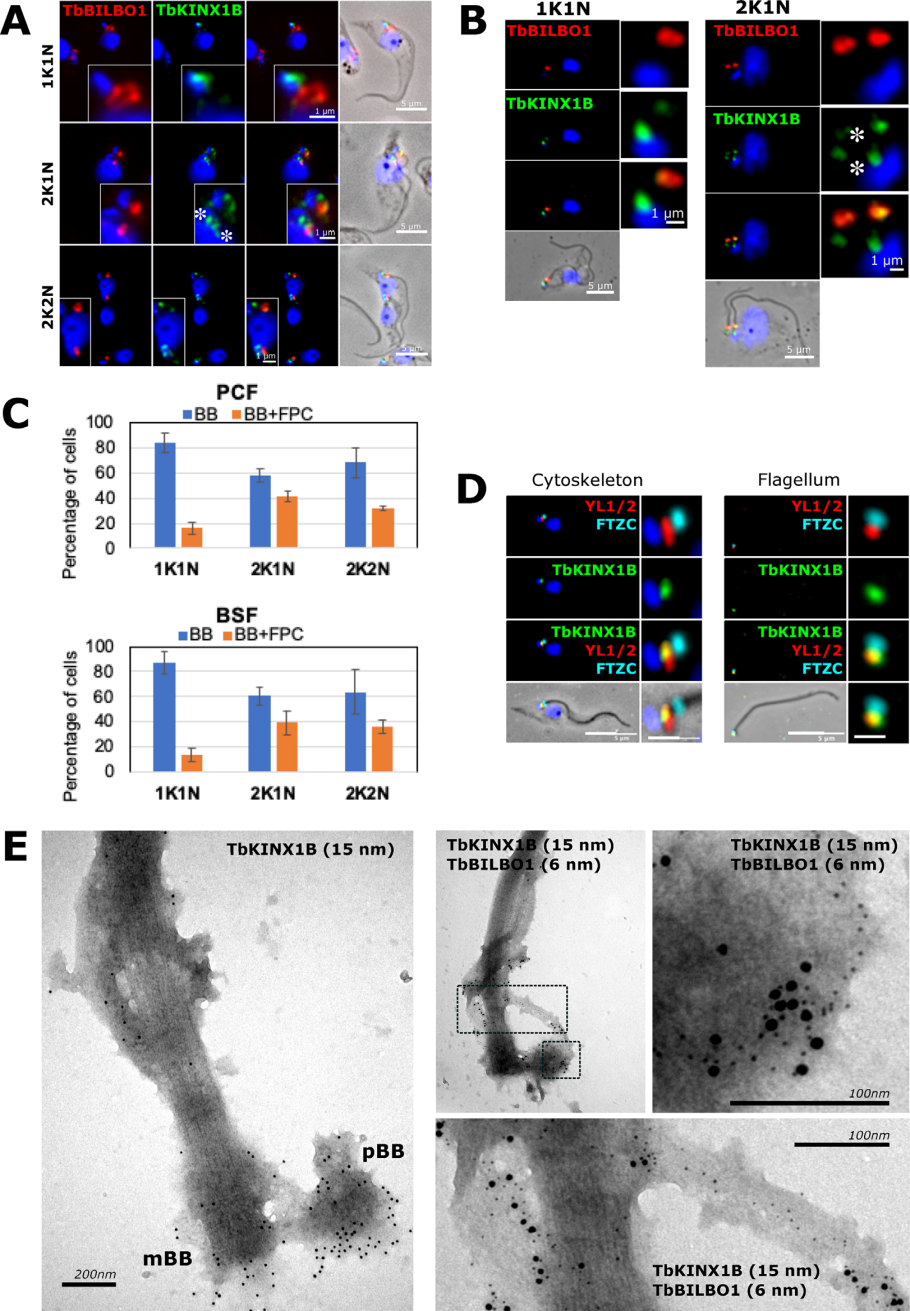
*TbKINX1B displays a dual localization.* A. Co-immunolabeling of TbKINX1B and TbBILBO1 on PCF detergent-extracted cells using anti-BILBO1 (red) and anti-TbKINX1B (green). B. Co-immunolabeling of TbKINX1B and BILBO1 on BSF detergent-extracted cells using anti-BILBO1 (red) and anti-TbKINX1B (green). C. Quantification of TbKINX1B BB and/BB+FPC localization in PCF and BSF from immunofluorescence experiments shown in A and B. BB localization (white bars) or BB and FPC localization (grey bars) were quantified at different cell cycle stages (200 cells, *n* = 3). D. Co-immunolabeling of TbKINX1B (green) and the basal bodies marker YL1/2 (red) and the transition zone marker FTZC (cyan) on detergent-extracted PCF cells cytoskeleton and flagellum. E. Immuno-TEM using PCF isolated flagella that were labelled with anti-TbKINX1B (15 nm gold particles), and co-immunolabeled with anti-TbKINX1B (15 nm gold) and anti-TbBILBO1 (6 nm gold). The images show positive labelling of the protein at the basal bodies (pBB, mBB), Flagellar Pocket Collar (FPC) and at the MTQ. The zoom image shows the TbBILBO1 6 nm gold beads on the MTQ and the colocalization of TbKINX1B and TbBILBO1. Scale bars in A, B and C represent 5 μm, and 1 μm in insets.

Many, but not all, kinesins move along microtubules and are therefore not strongly attached to the microtubule cytoskeleton. Western-blot analysis on whole cells (WC), detergent-extracted cytoskeleton (CSK) fraction and soluble fraction (S) of PCF and BSF using an anti-TbKINX1B, showed that TbKINX1B is expressed in both PCF and BSF *T. brucei* and almost quantitively associated with the cytoskeleton fraction, similar to TbBILBO1 (Fig. 1C). As TbKINX1B was identified as a binding partner of TbBILBO1 [21], we compared its subcellular localization to TbBILBO1 in PCF and BSF (Figs. 2A, 2B). We focussed on detergent extracted cytoskeletons which allow better visualization of the FPC and the basal body (BB) region. We localized TbKINX1B and TbBILBO1 using an indirect immunofluorescence assay (IFA) (Figs. 2A, 2B), with a combination of anti-TbKINX1B and anti-TbBILBO1 antibodies. In some cells, TbKINX1B was seen only in the BB region, whereas in other cells, a dual localization (BB and FPC) could be observed (asterisks). We quantified this localization pattern at different cell cycle stages (1 kinetoplast-1 nucleus 1K1N, 2 kinetoplasts-1 nucleus 2K1N, and 2 kinetoplasts-2 nuclei 2K2N) (Fig. 2C, 200 cells counted, *n* = 3). Our results showed that in 1K1N PCF and BSF cells, respectively, TbKINX1B is seen in the BB region only (blue bars) in 84% and 87% of the cells, and in the FPC and BB region (FPC + BB) (orange bars) in 16% and 13% of the cells. When cells progress through the cell cycle, the proportion of co-localization of TbKINX1B to FPC + BB doubles in cells that have duplicated their kinetoplast (42% and 39% in 2K1N cells and 32% and 36% in 2K2N cells). The basal body localization of TbKINX1B was further characterized using the marker FTZC, a transition zone protein [10] associated with the mature and pro-basal bodies (mBB and pBB), and the BB marker YL1/2 [30] (Fig. 2D). IFA was done on detergent extracted cytoskeletons and isolated flagella and showed good co-localization for TbKINX1B and YL1/2. We next determined TbKINX1B localization by immuno-electron microscopy analysis of isolated flagella and confirmed the localization on the BBs and the MTQ/FPC, where it co-localizes with TbBILBO1 (Fig. 2E). Taken together, these results show that TbKINX1B localizes at the BBs, the MTQ, and the FPC and that the localization of the pool of TbKINX1B appears to depend on the cell cycle stage, in both PCF and BSF cells.

### TbKINX1B and TbBILBO1 interact *in vivo* and B1BD is sufficient to target the FPC

**Figure 3:**
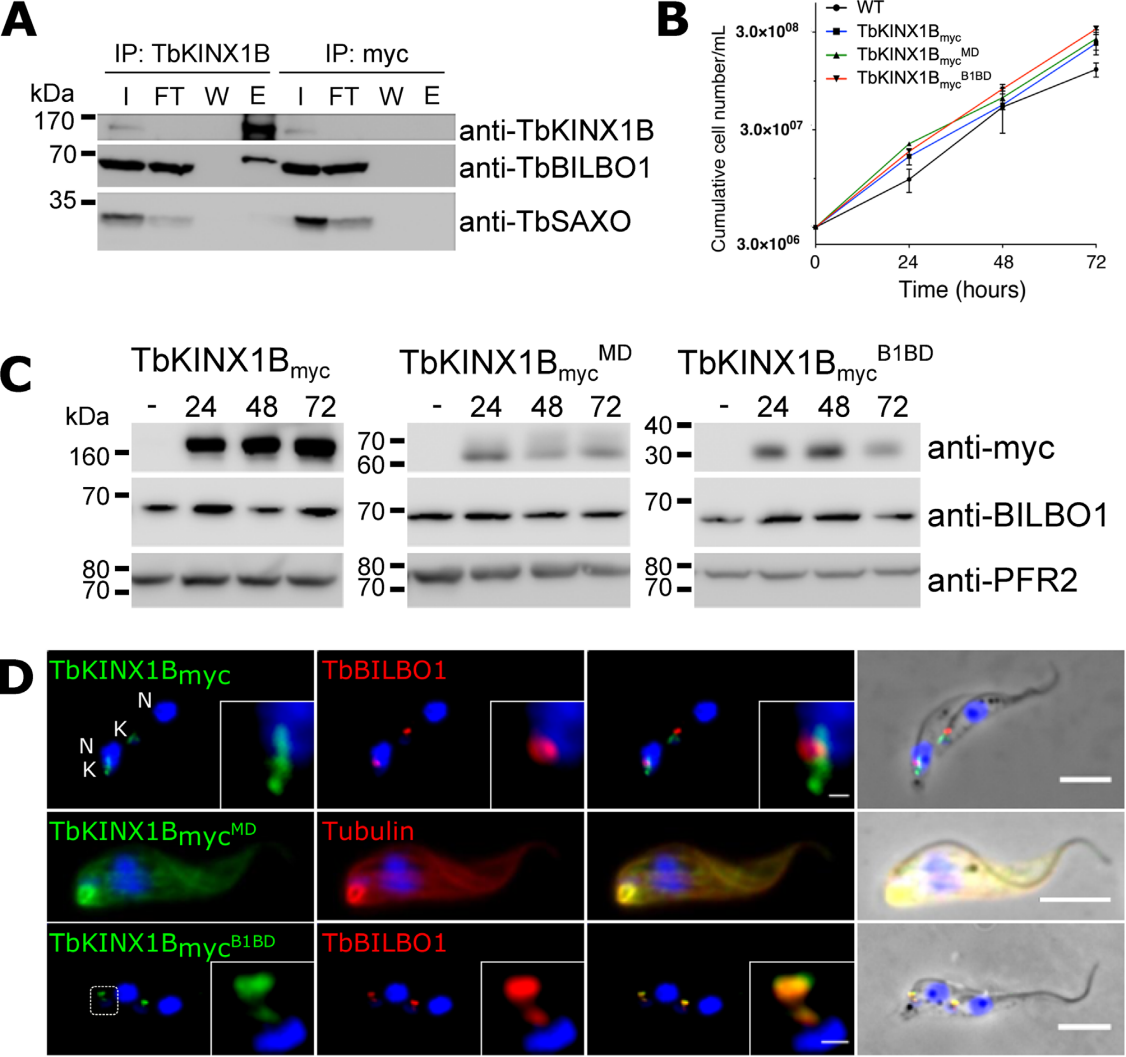
*Microtubule and FPC localization of TbKINX1B depends on its motor domain and BILBO1-binding domain respectively*. A. Immuno-precipitation of TbKINX1B and immuno-detection by western blotting of TbKINX1B and TbBILBO1 on input (I), flow-through (FT), wash (W), and elution (E) fractions. B. Induction of the expression of TbKINX1B_myc_, TbKINX1B_myc_^MD^ or TbKINX1B_myc_^B1BD^ does not affect PCF cell growth, as compared to WT (*n* = 3). C. Western blot of PCF WT cells and cells non-induced (–) or tetracycline-induced 24 h, 48 h, 72 h for the expression of TbKINX1B_myc_ (151 KDa), TbKINX1B_myc_^MD^ (62 KDa) or TbKINX1B_myc_^B1BD^ (28 KDa). Expressed proteins were detected using anti-myc antibody and using PFR2 (L8C4) as a loading control. No alteration on TbBILBO1 protein levels was observed. D. Co-immunolabelling on detergent-extracted cells of TbKINX1B_myc_ and TbKINX1B_myc_^B1BD^ proteins (green) with TbBILBO1 (red), and of TbKINX1B_myc_^MD^ (green) with Tubulin (red).

To test the TbKINX1B–TbBILBO1 interaction *in vivo*, we immunoprecipitated TbKINX1B from PCF cell extracts using a rabbit polyclonal antibody raised against the full-length TbKINX1B (Fig. 3A). Western blotting analysis showed that TbSAXO, an axonemal protein [17], is detected in the input samples (I) and the flow through (FT), but not in the elution sample (E). In contrast, TbKINX1B and TbBILBO1 are both detected in the input samples (I) and the elution sample (E) of the immunoprecipitation assay using anti-TbKINX1B, but not in the mock elution of the assay using a non-specific control anti-myc antibody. TbBILBO1 is thus specifically co-immunoprecipitated with TbKINX1B, suggesting that they belong to the same protein complex or interact directly. The latter possibility is strongly supported by the Y2H data that demonstrated the TbBILBO1–TbKINX1B B1BD interaction [21].

We next generated PCF cell lines that are inducible for the ectopic expression of a C-terminal myc-tagged version of full-length TbKINX1B (TbKINX1B_myc_, 156 KDa), the TbKINX1B motor domain (TbKINX1B_myc_^MD^, 62 KDa) or the TbKINX1B B1BD (TbKINX1B_myc_^B1BD^, 29 KDa) (Fig. 3). The expression of the constructs induced no growth defect over 3 days of induction (Fig. 3B) and did not affect the TbBILBO1 protein levels (Fig. 3C). TbKINX1B_myc_ was immuno-detected in the BB/FPC area, as previously observed for WT TbKINX1B (Fig. 3D). The motor domain localized to the MT cytoskeleton *in situ* as shown by co-labelling with an anti-tubulin antibody, supporting the MT-binding properties evidenced in the kinesin assay. In contrast, TbKINX1B_myc_^B1BD^ exclusively co-localized with TbBILBO1. This strongly suggests that the TbKINX1B BILBO1-binding domain is sufficient to target the protein to the FPC and to TbBILBO1. The expression levels of full-length TbKINX1B_myc_ tend to increase over time, whereas TbKINX1B_myc_^MD^ or TbKINX1B_myc_^B1BD^ protein levels appear to be reduced after 48 h and 72 h of induction. It is unclear why this is the case, but the expression of a truncated protein could be toxic or have cryptic and unwanted functions leading to down-regulation of expression or protein degradation.

### TbKINX1B is involved in basal body positioning in procyclic cells

**Figure 4:**
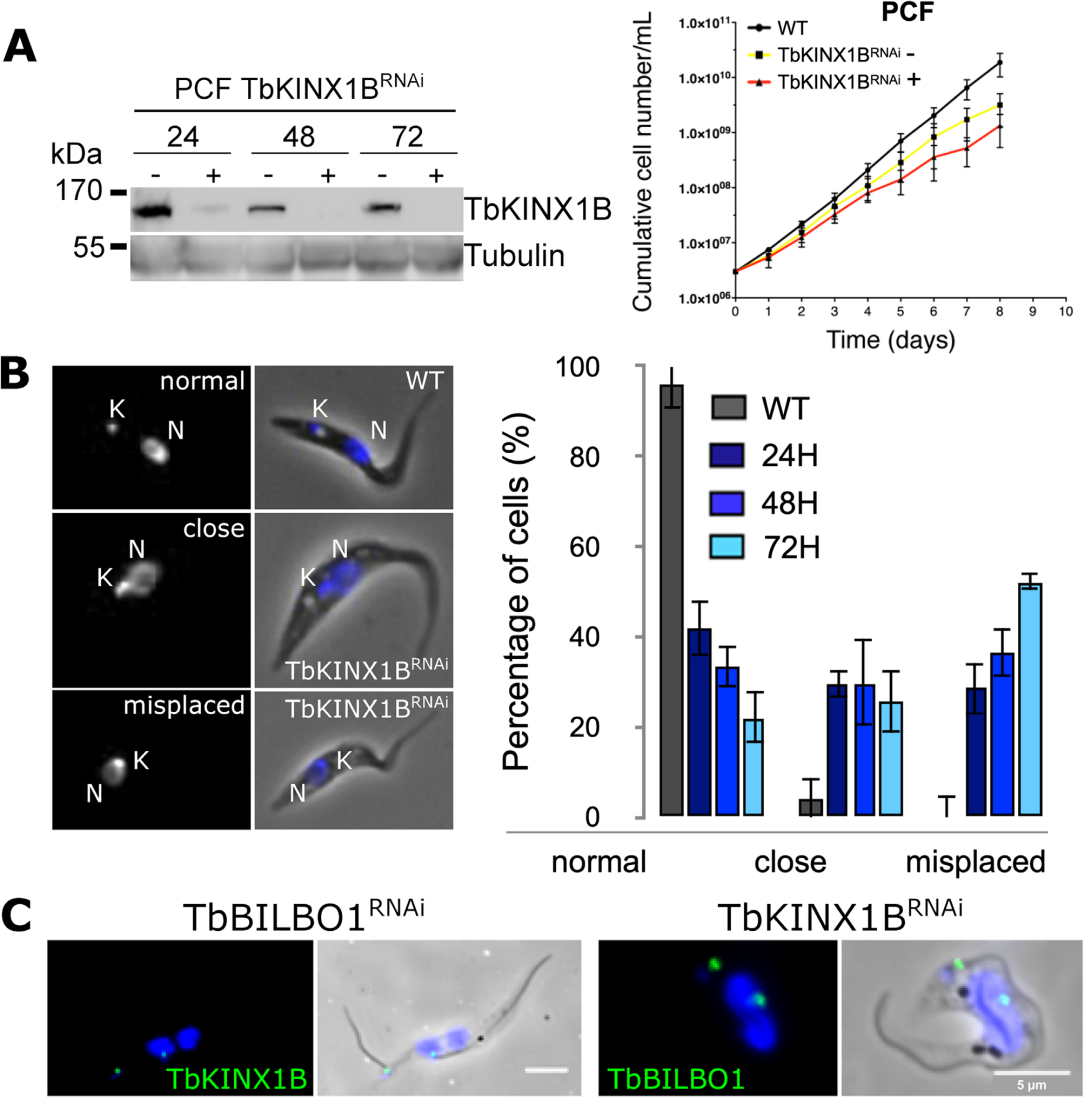
*TbKINX1B is involved in basal body/FPC segregation in PCF*. A. Left panel, Western blot analysis of whole cell protein extracts from PCF TbKINX1B^RNAi^ cell line in the presence (+) or absence (−) of tetracycline after 24 h, 48 h or 72 h probed with anti-TbKINX1B, anti-TbBILBO1, and anti-Tubulin (TAT1) as a loading control (left panel). Right panel, PCF growth curves showing that induction of RNAi down-regulation of TbKINX1B (TbKINX1B^RNAi^) does not dramatically affect cell growth when compared to WT and non-induced cells (*n* = 3). B. Down-regulation of TbKINX1B induces misplaced kinetoplasts. Left panel, IFA micrographs from CSK preparation of WT (a) and TbKINX1B^RNAi^ cell lines after 48 h of induction (b, c). Kinetoplasts (K) and nuclei (N) were stained with DAPI. Right panel, quantification of kinetoplast localization in 1K1N WT cells and in TbKINX1B^RNAi^ cells after 24 h, 48 h and 72 h of induction. C. Left panel, immunolocalization of TbKINX1B (green) in TbBILBO1^RNAi^ induced detergent-extracted cells. Right panel, immunolocalization of TbBILBO1 (green) in *TbKINX1B*^RNAi^ induced detergent-extracted cells. Scale bars 5 μm.

To elucidate the function of TbKINX1B in *T. brucei*, we characterized the effect of its down-regulation in PCF using tetracycline-inducible RNA interference (RNAi) [3] (Fig. 4). Immunoblotting confirmed that TbKINX1B protein levels were reduced after 24 h of induction and undetectable after a longer induction time (Fig. 4A, left panel). After RNAi induction, a significant reduction of the growth rate was observed (Fig. 4A, right panel).

Light microscopy observation of PCF WT and TbKINX1B^RNAi^ cells after 24 h to 72 h knockdown revealed a small subset of the population with an abnormal number of kinetoplasts (K) or nuclei (N) and FPC, and/or with detached and multiple flagella (Fig. S2A). In these cells, the absence of FAZ detection at the new detached flagella suggests a FAZ assembly defect leading to flagella detachment from the cell body. The newly detached flagellum was often found located close to the old mother flagellum, suggesting a basal body segregation defect (Fig. S2A). All these abnormal cells represent less than 10% of the population and are unlikely to have a significant effect on population growth within the observed period. TbKINX1B^RNAi^ cells, after 24 h RNAi induction, display an abnormal positioning of the kinetoplast phenotype as shown by DAPI staining (Fig. 4B, graph). Quantification of kinetoplast positioning, in 1K1N cells, with reference to the nuclei (N) indicated that after 24 h induction, the spatial organization of the K within the population had changed and consisted of three groups (Fig. 4B). The first was “normal” (41.7%) whereby the K was located in the posterior of the cell as in WT 1K1N cells. The second was “close” (29.5%), in which the K was at the posterior of the cell but close to the nucleus. The third was “misplaced” (28.7%), where the K was located close to the nucleus but had relocated to the periphery of the cell in between the nucleus and the sub-pellicular MTs or anterior to the nucleus. The percentage of these subsets of cells continued to increase over the period of 48 h to 72 h RNAi induction, eventually becoming the dominant sub-populations, with the abnormal phenotypes “close” and “misplaced” reaching 78%. These results suggest that kinetoplast positioning is affected as a direct or indirect consequence of TbKINX1B depletion in PCF. Interestingly, TbKINX1B signal was localized to the BB of the detached new flagellum in TbBILBO1^RNAi^-induced cells suggesting that BB localization of TbKINX1B does not depend on TbBILBO1 (Fig. 4C). The converse is also true as TbBILBO1 localization and expression were not affected in TbKINX1B^RNAi^-induced cells (Figs. 4C, S2).

### TbKINX1B is essential in bloodstream cells

**Figure 5:**
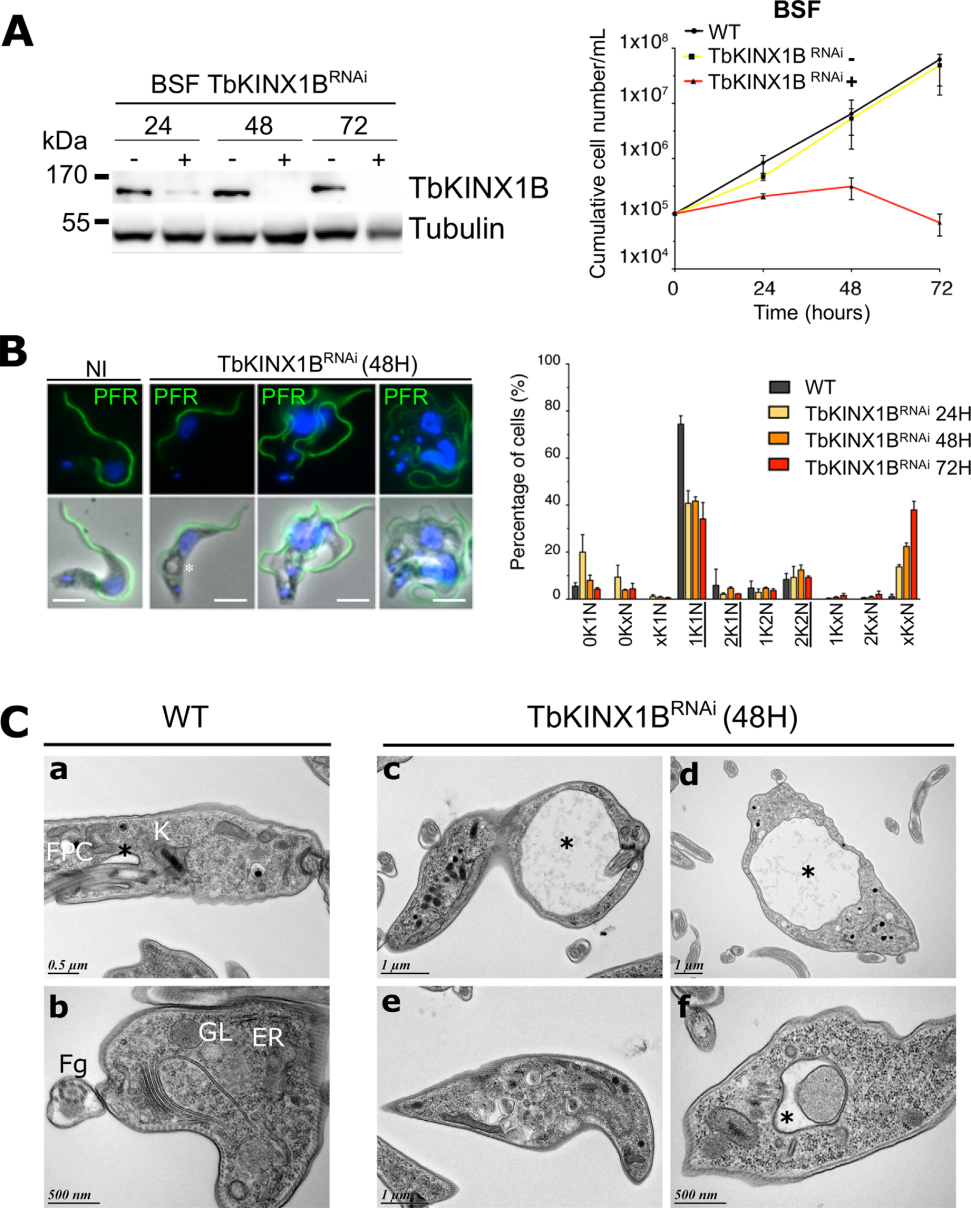
*TbKINX1B is essential in bloodstream forms*. A. Left panel, western blot of BSF whole cell protein extracts from TbKINX1B^RNAi^ cell line in the absence (−) or presence (+) of tetracycline at 24 h, 48 h and 72 h using anti-TbKINX1B, and anti-tubulin (TAT1) as a loading control. Right panel, down-regulation of TbKINX1B in BSF (+) inhibits cell growth after 48 h of induction, as compared to non-induced cells (−) (*n* = 3). B. Left panel, IFA micrographs of WT and of 48 h induced TbKINX1B^RNAi^ BSF cell lines. Immunofluorescence shows that down-regulation of TbKINX1B leads to multi-flagellated and multinucleated cells. Flagella were labelled with anti-PFR2 (green), and kinetoplasts and nuclei were DAPI-stained (blue). A large FP or vacuole is marked with an asterisk. Scale bars 5 μm. Right panel, quantification of cell division phenotypes in WT and in TbKINX1B^RNAi^ cell lines after 24 h, 48 h and 72 h of induction. Normal phenotypes are underlined (200 cells, *n* = 3). C. Transmission Electron Microscopy (TEM) thin-sections of WT BSF and of TbKINX1B^RNAi^ induced for 48 h. The structural organization of WT cells reveal well-defined FP (black asterisk), FPC, Golgi (G), endoplasmic reticulum (ER), recycling endosomes (RE), glycosomes (GL), Flagellum (Fg), internal vesicles and kinetoplast (K) (a, b). TbKINX1B knock-down cells possess enlarged FP (black asterisk, c, d), with the disturbed endo-membrane organization (i.e., FP harboring dense material or enlarged) (e, f).

We further investigated the consequences of *TbKINX1B* RNAi down-regulation (TbKINX1B^RNAi^) in BSF (Fig. 5). Quantification of BSF immunoblots (*n* = 3) probed with anti-TbKINX1B indicated that after 24 h of RNAi induction, TbKINX1B levels had decreased by 60% as compared to non-induced cells, and after 48 h, only 24% could be detected (Fig. 5A). Very shortly after induction, a growth defect was observed, leading to growth arrest after 24 h of induction (Fig. 5A, graph). Immuno-labelling of the flagellum, using an anti-paraflagellar rod antibody, and DAPI staining showed that RNAi-induced cells displayed an abnormal number of K and N after 24 h of induction, associated with multi-flagellated phenotypes such as 2K and 4 flagella (Fig. 5B), suggesting a defect in cell division. Quantification (*n* = 3) of the different cell populations showed an increase of xKxN cells over a period from 24 h to 72 h, corresponding to 14% and 38%, respectively (Fig. 5B, bar graph). Finally, TbKINX1B^RNAi^-induced cells exhibited an enlarged flagellar pocket phenotype which appears to be similar to the “Big Eye” phenotype first observed after RNAi knockdown of clathrin in BSF, leading to a defect in endocytosis but not exocytosis [2]. This TbKINX1B^RNAi^-induced “Big Eye” also suggests several rounds of unsuccessful endocytosis, or an imbalance between endocytosis and exocytosis rates. This is also accompanied by numerous abnormal cytoplasmic vesicles, and abnormal material within the FP, as observed by electron microscopy on thin sections of whole cells (Fig. 5C-f).

## Discussion

*TbKINX1B* (Tb927.7.3000) is a putative N-kinesin that belongs to the kinesin group X1, a small group of kinesins present only in the kinetoplastids [44, 5]. Using the TriTryDB BlastP tool, TbKINX1B orthologs were found in the parasites represented by *Trypanosoma* and *Leishmania*, but also the neotropical porcupine parasite *Porcisia hertigi* (34% aa identity), *Leptomonas seymouri* (40% aa identity), the monoxenous parasites *Crithidia fasciculata* (41% aa identity), *Blechomonas ayalai* (44% aa identity), *Paratrypanosoma confusum* (38% aa identity), and in the free living kinetoplastid *Bodo saltans* (45% aa identity) [6].

The protein appears to localize to some degree along the MTQ in both PCF and BSF. This localization is differentially distributed according to the cell cycle stage with the BB + FPC signals being more abundant in 2K1N cells. This variation is probably not related to expression levels as this does not change during the cell cycle [16] and this may reflect the importance of the protein in the stages where organelle division and segregation are most important [26, 38]. Kinesin motor domains are predominately associated with microtubules; their interaction with adaptor and scaffold proteins may define the type of cargo to transport and thus its function in intracellular transport. Previous FP structural analysis in *T. brucei* by electron tomography freeze-fracture shows that the endocytic markers (fluid or receptor-mediated) are located at the FP membrane, at the posterior and anterior face, except in the region directly associated with the MTQ, the neck MT, and the axoneme [22]. It has also been demonstrated that when internalization is blocked, in BSF, endocytic markers accumulate in a channel that runs the length of the neck and is closely associated with the MTQ, by which extracellular components can gain access to the FP lumen [22]. If we consider the motor domain homology of TbKINX1B to KIF3A (a well-studied mammalian kinesin), it suggests that the protein associates with microtubules that are linked to endosomes [7] or with recycling endosomal tubules [19]. The general endomembrane network disorganization in BSF *Tb*KINX1B^*RNAi*^ cells observed by EM suggests that TbKINX1B may play a role in intracellular trafficking, which is extremely important in BSF.

### TbKINX1B is a TbBILBO1 protein partner

Immuno-electron labelling of TbKINX1B also revealed a mature and immature basal body localization. Labelling was also observed on the FPC where it colocalizes with TbBILBO1. This co-localization suggests that BILBO1 could interact with TbKINX1B as a cargo. This is supported by the immunoprecipitation assays (Fig. 3A) and the Y2H assays with a direct interaction between TbKINX1B B1BD and TbBILBO1 [21]. Further, both the TbKINX1B B1BD and the BILBO1 calcium-binding EF-hands domains are necessary for the interaction. Interestingly, TbBILBO1 EF-hands are also involved in the interaction with the newly identified FPC protein TbBILBO2 [28]. However, unlike BILBO2 [21], the mutation of both EF-hands 1 and 2 of BILBO1 abolished the interaction with TbKINX1B. This suggests that TbKINX1B interaction with TbBILBO1 could depend on the calcium loading status of the TbBILBO1 EF-hands, unlike the interaction between the CaBP2933 EF-hands and kinesin-3 in *Giardia intestinalis* where binding was not calcium dependent [4].

Because TbKINX1B and TbBILBO1 physically interact, it was surprising to observe that knockdown of TbKINX1B or overexpression of its B1BD did not affect the localization of TbBILBO1, suggesting that TbBILBO1 is not an *in vivo* TbKINX1B cargo. This could be due to numerous reasons such as incomplete depletion of TbKINX1B by RNAi, temporally limited interaction or the requirement of numerous co-factors. Additionally, for each cargo, there may be more than one motor protein, which could indicate functional redundancy as previously described [8, 14, 43]. Indeed, among the 28 identified *T. brucei* kinesin genes [44], at least 7 (Tb927.3.2040, Tb927.6.1770 (KIN-G), Tb927.6.2880, Tb927.7.3000, Tb927.8.4840, Tb927.11.5300 (KIN13-3), and Tb11.v5.0819) are localized at the BB-FPC area by the TrypTag Genome-wide Protein Localisation Resource [18]. One could imagine that in the absence of TbKINX1B, TbBILBO1, which is an essential protein, could be transported by a different, not yet identified, kinesin.

### TbKINX1B is not essential in PCF but is vital in BSF

Long-term knockdown of *TbKINX1B* in PCF parasites generated BBs positioning defect, with 60% of the population showing kinetoplast positioning different to WT cells. Since the phenotype was not lethal, it suggests either that in PCF *TbKINX1B* has a minor function, or that RNAi was incomplete and can be compensated by other kinesins, or that the kinetoplast mis-positioning may be a downstream effect. Since PCF and BSF separate their basal bodies slightly differently, the role of TbKINX1B in basal body placement may be more important in BSF. It is also possible that TbKINX1B has a completely different role in BSF cells and that this function is disrupted once the protein is depleted. Overexpression of different *TbKINX1B* domains did not induce dominant-negative phenotypes. However, it did show that TbKINX1B^B1BD^ localizes only to the FPC, suggesting that TbKINX1B binds directly to BILBO1 *in vivo*, supporting the Y2H and IP data. Furthermore, and as expected for a kinesin, the TbKINX1B motor domain co-localizes on MTs *in vivo*. It is thus possible that by simultaneously binding to BILBO1 and microtubules, TbKINX1B could transport BILBO1 along MTs.

Depletion of *Tb*KIN-C in PCF induced a basal body segregation defect and interrupted correct cytokinesis, eventually leading to cell death [27]. Interestingly, *Tb*KIN-C can also influence protein expression of the orphan kinesin *Tb*KIN-D [43]. Thus, TbKINX1B could also influence the expression of other kinesins and/or cell cycle proteins that can eventually compensate for TbKINX1B (including different family groups), or *vice-versa*, and thus also produce secondary phenotypes related to cell division in PCF.

Clearly, TbKINX1B has a more important role in BSF. BSF depletion of TbKINX1B resulted in growth arrest after 24 h and proved to be lethal within 72 h. Cells were unable to divide correctly and displayed enlarged flagellar pockets, a phenotype known as “Big Eye”. This phenotype has been previously observed in mutants with defects in endo- or exocytosis. Interestingly, it has also been observed in RNAi knockdown of some proteins belonging to the cytoskeleton, such as MORN1 and BHALIN. Here it is thought the “Big Eye” outcome is a downstream effect of interference in the structure or function of the flagellar pocket or components of that region [2, 11, 23, 35]. It is unclear whether TbKINX1B “Big Eye” phenotypes are due to direct influence on endocytosis or due to a more general disruption of the flagellar pocket region. The difference between the phenotypes produced in PCF and BSF could be due to the efficiency of RNAi, but also to the shorter doubling time in BSF and the increased rate of endo- and exocytosis in BSF compared to PCF (for a recent review, see [31]). However, the importance of the more rapid endo-exocytotic system in BSF is clearly emphasized by the lethality induced by TbKINX1B RNAi.

It is important to note that the cargos for TbKINX1B, if any, remain unknown. Furthermore, TbKINX1B and BILBO1 interact *in vivo* but there is no evidence of BILBO1 localization being affected by depletion of the TbKINX1B in PCF and BSF. This is especially interesting in BSF given the dramatic RNAi phenotype observed. This may be explained if TbKINX1B has a structural role in BSF rather than transporting cargos; its knockdown would lead, similarly to MORN1 and BHALIN RNAi, to the Big Eye phenotype in BSF.

In conclusion, we have described the localization, the essentiality, and the plausible functional roles of the protein TbKINX1B in *T. brucei*. Further experiments will be necessary to understand the extent of TbKINX1B function, but our data suggest that TbKINX1B and TbBILBO1 are, during a restricted temporal period, at least part of a protein complex.

## Conflict of interest

The authors declare that they have no competing interests.

## Authors’ contributions

DP, EB, GL-K, NL, DD and AS performed the experiments, AC and DRR performed the EM experiments, DP, KE, LK and DRR designed the experiments. DP, MB, LK, KE and DR wrote the manuscript.

## Funding

This work was funded by the Centre National de la Recherche Scientifique (CNRS), the University of Bordeaux, the Aquitaine Regional Council Grant – 20111301014, the ANR (ANR-09-BLAN-0074 and ANR PRCI ANR-20-CE91-0003), and the Laboratoire d’Excellence (LabEx) ParaFrap grant (ANR-11-LABX-0024). DP was a postdoc recipient from the LabEx ParaFrap. The electron microscopy was done in the Bordeaux Imaging Center, a service unit of the CNRS-INSERM and Bordeaux University, member of the national infrastructure France BioImaging supported by the French National Research Agency (ANR-10-INBS-04).

## Supporting information

The Supplementary materials of this article are available at https://www.parasite-journal.org/10.1051/parasite/2022015/olm**Figure S1:**
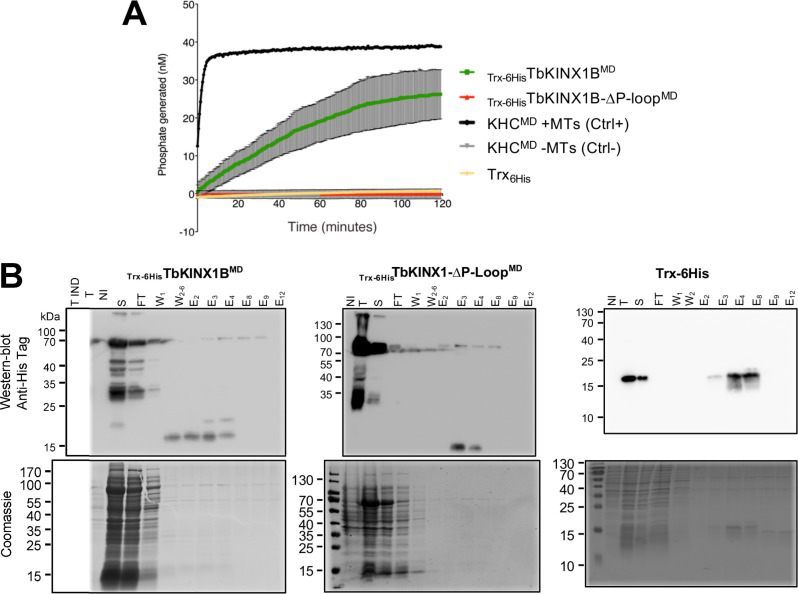
*TbKINX1B can hydrolyse ATP* in vitro. A. ATPase activity using the ELIPA *in vitro* assay. Kinesin activity was measured by the amount of generated phosphate per minute, in the presence of taxol-stabilized microtubules and ATP. The human kinesin heavy chain motor domain (KHC^MD^) is the positive control kinesin in presence of MTs (+MTs, black line) or negative control in the absence of MT (−MTs, grey line), the TbKINX1B^MD^ (green), TbKINX1B^MD^ deleted for the ATPase domain (ΔP-loop^MD^, red line), and _6His_TRX purified protein (yellow line), (*n* = 3). B. Recombinant TbKINX1B proteins purification. Western blot analysis (upper panel) and Coomassie-stained SDS-PAGE (lower panel) of the purification of the _Trx-6His_TbKINX1B^MD^ (A), _Trx-6His_TbKINX1B-ΔP-loop^MD^ (B), and Trx-6His (C) proteins. The recombinant proteins were immunolabelled using anti-Histidine tag. Abbreviations: Total (T), non-induced (NI), induced (IND), supernatant (S), flow-through (FT), wash (W, different wash fractions numbered), Elution (E, fractions numbered).**Figure S2:**
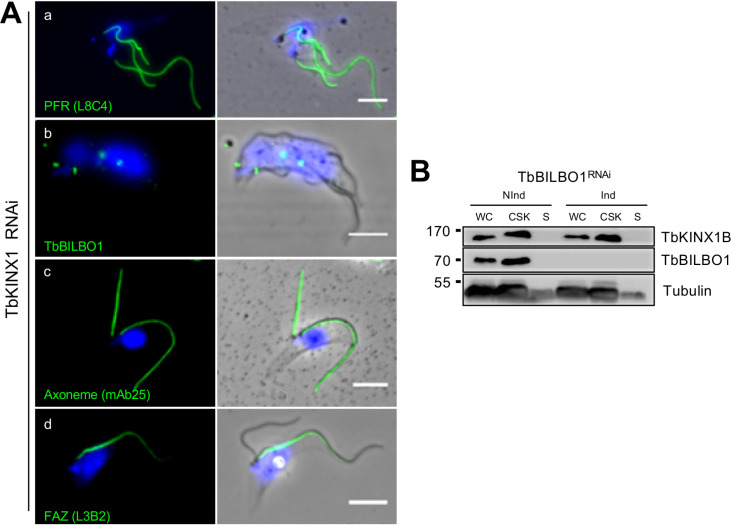
*Impact of RNAi knockdown of TbKINX1B or TbBILBO1 in PCF on expression and localization*. A. Minor phenotypes were observed in TbKINX1B^RNAi^ cells induced for 24 h to 72 h. (a) The flagella were labelled with anti-PFR antibody (L8C4). (b) The FPC structures were labelled with anti-TbBILBO1 antibody. (c) The axonemes were labelled with anti-TbSAXO antibody (mAb25). (d) The FAZ structure was labelled with the anti-FAZ antibody (L3B2). Scale bars 5 μm. B. Expression level of TbKINX1B upon RNAi down-regulation of TbBILBO1. Immunoblotting of TbKINX1B, TbBILBO1 and Tubulin in whole cell protein extracts (WC), detergent-extracted cytoskeleton fraction (CSK), and soluble fraction (S) of lysed TbBILBO1^RNAi^ non-induced (NInd) or induced for 36H (Ind) cells.
